# Hedgehog Signaling and Embryonic Craniofacial Disorders

**DOI:** 10.3390/jdb7020009

**Published:** 2019-04-24

**Authors:** John Abramyan

**Affiliations:** Department of Natural Sciences, University of Michigan-Dearborn, Dearborn, MI 48128, USA; abramyan@umich.edu

**Keywords:** Hedgehog, craniofacial, *Shh*, *Ihh*, holoprosencephaly, ciliopathy

## Abstract

Since its initial discovery in a *Drosophila* mutagenesis screen, the Hedgehog pathway has been revealed to be instrumental in the proper development of the vertebrate face. Vertebrates possess three hedgehog paralogs: Sonic hedgehog (*Shh*), Indian hedgehog (*Ihh*), and Desert hedgehog (*Dhh*). Of the three, *Shh* has the broadest range of functions both in the face and elsewhere in the embryo, while *Ihh* and *Dhh* play more limited roles. The Hedgehog pathway is instrumental from the period of prechordal plate formation early in the embryo, until the fusion of the lip and secondary palate, which complete the major patterning events of the face. Disruption of Hedgehog signaling results in an array of developmental disorders in the face, ranging from minor alterations in the distance between the eyes to more serious conditions such as severe clefting of the lip and palate. Despite its critical role, Hedgehog signaling seems to be disrupted through a number of mechanisms that may either be direct, as in mutation of a downstream target of the Hedgehog ligand, or indirect, such as mutation in a ciliary protein that is otherwise seemingly unrelated to the Hedgehog pathway. A number of teratogens such as alcohol, statins and steroidal alkaloids also disrupt key aspects of Hedgehog signal transduction, leading to developmental defects that are similar, if not identical, to those of Hedgehog pathway mutations. The aim of this review is to highlight the variety of roles that Hedgehog signaling plays in developmental disorders of the vertebrate face.

## 1. Introduction

Developmental disorders of the craniofacial region are among the most common birth defects in humans (cleft lip and palate have a prevalence of ~1:700 births [1,2]), and the Hedgehog (Hh) signaling pathway is often intimately involved. Hh signaling is a versatile tool for development and can function in short-range, long-range, direct, indirect, and concentration-dependent manner; allowing for adjustable response based on signal threshold, as well as spatiotemporal expression [3]. In vertebrates, the Hh pathway has long been implicated in a number of roles during embryonic development, including neural crest cell survival [4], left-right asymmetry [5,6], anteroposterior patterning of limbs [7,8], dorsoventral patterning of somites [9], as well as the development of the eyes [10,11,12], bone [13], cartilage [14,15], gonads and germ cells [16], muscle [17,18,19,20], nervous system [21,22,23], and teeth [24,25]. Ingham and McMahon (2001) have compiled a comprehensive list demonstrating the diversity of Hh pathway functions [3]. 

In vertebrates, the *Drosophila* hedgehog gene has evolved into three paralogs: Sonic hedgehog (*Shh*), Indian hedgehog (*Ihh*), and Desert hedgehog (*Dhh*) [21,26]. Of the three, *Shh* is the most broadly expressed and is responsible for the majority of Hh function during craniofacial development [27,28,29,30,31], from the survival of newly migrated cranial neural crest cells [4] to fusion of craniofacial prominences [30,32]. *Ihh* has a more limited role during embryogenesis, mainly known for regulating bone development [15,33]. *Dhh* is also very limited in its expression, primarily consigned to the male reproductive tract and germ line [16,34], although expression has been detected in the peripheral nervous system [35,36]. Thus, expression patterns and functions differ spatiotemporally across the three vertebrate Hh paralogs.

## 2. The Discovery and Evolution of Hedgehog Genes

The hedgehog gene was originally discovered in *Drosophila melanogaster*. During a mutagenesis screen, Nüsslein-Volhard and Wieschaus identified a mutant in which the pattern of denticles on the ventral cuticle was disrupted [37]. Normally, denticles are consigned to the anterior of each segment, while the posterior remains as naked cuticle. In the hedgehog mutant, bristles appeared across the entire segment. Subsequent studies identified the hedgehog gene as the likely culprit, confined to cells in the posterior compartment of each segment primordium, overlapping with disrupted bristle patterning [38,39,40].

Following identification in *Drosophila*, the three hedgehog orthologs were characterized in vertebrates in initial studies led by Andrew McMahon (mouse [21]), Philip Ingham (zebrafish [41]), and Cliff Tabin (chicken [7]). Subsequent reports further detailed the role of the vertebrate Hh genes in floor plate induction and limb patterning, as well as detailing its molecular processing [22,23,42]. *Ihh* and *Shh* are more closely related to each other and likely arose from a more recent duplication event after their ancestor gene diverged from *Dhh* [26]. Teleost fish have additional Hh genes due to their additional genome duplication, giving rise to duplicates of *Shh* (known as *shhb* or tiggywinkle hedgehog *(twhh)* [43]) and *Ihh* (known as *Ihha* [44] or echidna hedgehog (*ehh*) [45]). These studies initiated decades of subsequent research in an attempt to understand the various roles that the Hh pathway plays in vibrate embryology, a colossal task that continues to this day.

## 3. The Hedgehog Pathway

**Figure 1:**
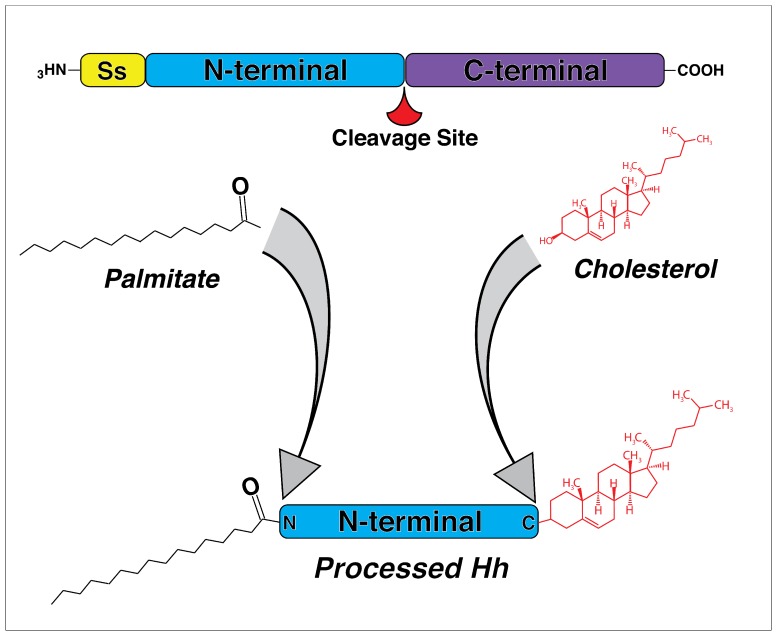
Biogenesis of processed Hedgehog (Hh) ligand. Hh ligand is produced as a large precursor protein that undergoes a series of post-translational modifications prior to secretion. The N-terminal signaling domain is first cleaved from the C-terminal processing domain. Subsequent to cleavage, the N-terminal has a palmitate added to its N terminus and cholesterol added to its C terminus, resulting in a biologically active, processed Hh ligand that may be transported out of the cell. Ss—Signal sequence. Adapted from reference [56].

In both vertebrates and invertebrates, Hh signaling shares a similar signal transduction cascade where an activated ligand leaves the cell and diffuses through extracellular space, acting as a concentration-dependent morphogen that binds to downstream receptors [46,47,48]. The Hh ligand is initially synthesized as large a precursor and has to undergo post-translational modification before becoming a functional molecule. The precursor first undergoes autocatalytic cleavage into N- and C-terminal fragments, with biological activity consigned to the N-terminal [49,50,51] (Figure 1). The C-terminal fragment catalyzes the cleavage and cholesterol transfer reactions required for modification of the N-terminal [51]. The N-terminal fragment undergoes a covalent lipid modification through the addition of cholesterol and palmitate [52] (Figure 1). Modification with lipid moieties is thought to allow Hh ligands to associate with lipid rafts in the plasma membrane [53], as well as facilitate multimeric complex formation during signal transduction, which likely increases activity [54,55]. Thus, cleavage and subsequent post-translational modification are critical for the production of an active ligand that can ultimately leave the cell.

**Figure 2:**
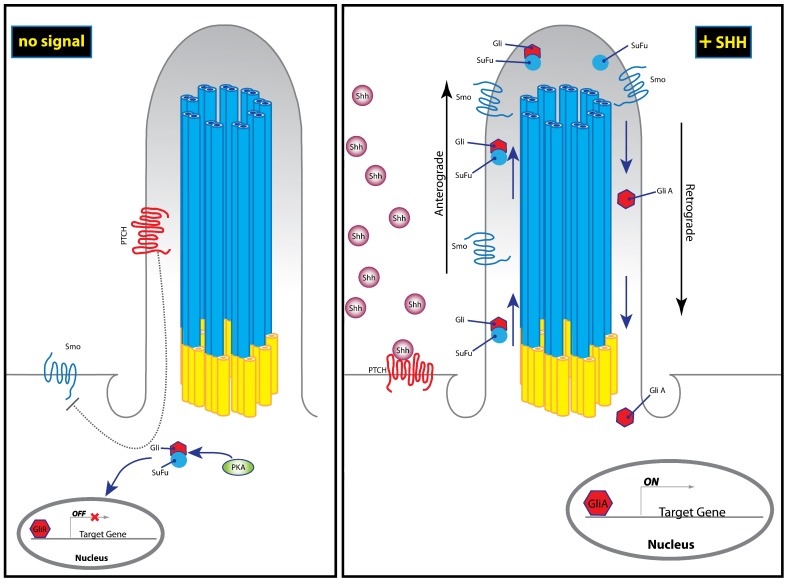
In the absence of Hh signal, Patched1 (*Ptch1)* inhibits the Hh pathway through the modulation of the Smoothened *(Smo)*. Since *Smo* is inactivated, the Glioma-associated oncogene- Suppressor of Fused (*Gli*-*Sufu)* complex is retained in the cytosol, where it is exposed to phosphorylation by protein kinase A (PKA), thereby inhibiting signal transduction by *Gli*. When Hh signal is present (here represented by *Shh*), its binding to *Ptch*1 relives pathway inhibition, induces its translocation away from the cilium, allowing activated *Smo* and *Gli*-*Sufu* complex transport to the tip of the cilium, where *Gli* becomes dissociated from *Sufu*. Subsequent retrograde transport of *Gli* then brings it to the nucleus, where it regulates expression of target genes. Image adapted from Reference [76,77].

Once associated with the plasma membrane, the activated Hh ligands are released from the cell through the activity of two proteins: Dispatched (*Disp*) [47,48,57] and Signal Peptide CUB EGF-like domain-containing protein (*Scube2*) [48,58]. Subsequent Hh pathway transduction occurs in target cells—both in short and long range—by direct binding of the Hh ligand to Patched1 (*Ptch1*), a twelve-pass transmembrane domain receptor [59,60,61]. In the absence of ligand binding, *Ptch1* constitutively inhibits activity of the Hh pathway through the modulation of Smoothened (*Smo*) [61,62]. *Smo* is a G-protein coupled receptor (GPCR)-like protein that functions as a mediator of intracellular Hh signal transduction within the primary cilium [63] (Figure 2). Hh binding is thought to relieve inhibition of *Smo* by *Ptch1*, thereby allowing Hh signal transduction to occur [64,65]. Hh binding is also thought to induce changes in *Smo* phosphorylation state, cellular localization, and protein conformation [66,67]. Authors have proposed that binding to *Ptch1* may also serve to sequester the Hh protein and limit its diffusion, thereby controlling the spatiotemporal range of activity [64]. Sequestration of Hh by *Ptch1* may also then facilitate re-establishment of quiescence in the responding cell [64,68,69]. These early steps define the direct function of the Hh ligand after it leaves the cell: facilitating the activation of *Smo* through binding to *Ptch1*.

The mechanism of *Smo* inhibition by *Ptch1* remained a topic of debate for a period of time and was thought to involve a small molecule intermediate since *Ptch1* is structurally related to proteins that act as small molecule pumps [62,70]. Several authors initially proposed that these intermediate molecules might be sterols [71,72]. Corcoran and Scott, (2006) describe *Ptch1* as pumping oxysterols away from *Smo*, thereby inhibiting *Smo* activation until the Hh ligand binds to it and stops the pumping action [71]. More recent work however, showed that cholesterol itself binds and activates *Smo*, as opposed to oxysterols [73,74]. Recent work on the structure of *Ptch1* has also begun to confirm its role as a pump for cholesterols that is blocked by the Hh ligand upon binding [75].

Hh binding to *Ptch*1 then relieves *Smo* inhibition and allows active *Smo* to accumulate in the distal tip of the primary cilium in order to mediate downstream signaling [63,78,79]. It then dislodges the Glioma-associated oncogene (*Gli*) family of transcription factors (*Gli*, *Gli2*, and *Gli3*) from a complex involving Suppressor of Fused (*Sufu*) [80]. *Sufu* is a negative regulator of Hh signaling through binding and sequestration of *Gli* proteins [81,82,83]. The *Gli*-*Sufu* complex also facilitates retention of *Gli* in the cytosol, which exposes it to phosphorylation by protein kinase A [84], inhibiting downstream signal transduction. However, upon activation of *Smo*, *Gli2/3*-*Sufu* complex is dismantled, allowing retrograde transport of *Gli* in order bring it back to the nucleus, where it regulates expression of target genes [78] (Figure 2).

Both *Gli2* and *Gli3* can be found in long activator form (GliA) or can be proteolytically cleaved to a truncated repressor form (GliR). In vitro, both *Gli2*R and *Gli3*R are able to inhibit Hh pathway genes. However, to date, the majority of evidence suggests that *Gli3* is primarily the repressor while *Gli2* is primarily the activator of the signaling pathway [85]. Our current understanding of *Gli* processing in the cilium suggests that recruitment of *Smo* to the cilium leads to an increase in the ratio of GliA:GiR [86]. Furthermore, different tissues appear to use different ratios of GliA:GliR, suggesting a complex interplay between the various forms of GLI protein and three *Gli* paralogs (detailed by Liu, 2016 [85]). Taken together, these studies have delineated a complex pathway of protein interactions that produce very similar phenotypes if disrupted; greatly complicating attempts at identifying the causes of Hh associated disorders due to a mutation in one member of this pathway. 

## 4. Craniofacial Development 

**Figure 3:**
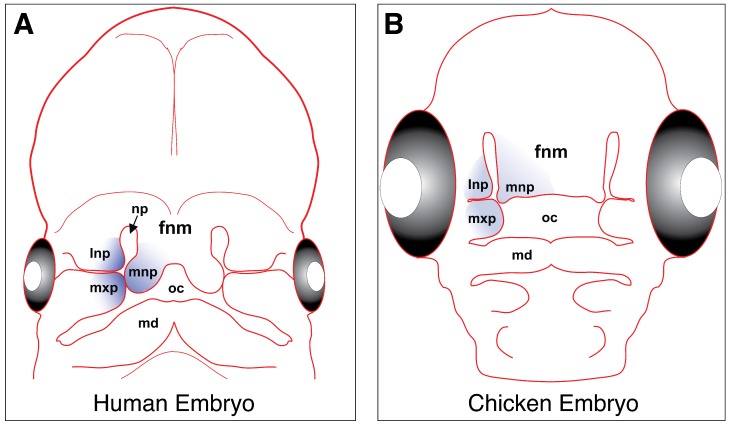
Fusion of the upper lip in human (**A**) and chicken (**B**) embryos reveals structural similarities. Across amniotes, the face is assembled from five main craniofacial prominences: the frontonasal mass (fnm), lateral nasal prominence (lnp), medial nasal prominence (mnp), maxillary prominences (mxp) and the mandibular prominences (md). The primary palate (upper lip/beak and nasal cavities/nasal pits [np]) forms from the fusion of the maxillary and lateral nasal prominences with the medial nasal prominence (highlighted in blue), although there is some variation in this process across different lineages [92].

Craniofacial development is initiated by cranial neural crest cells; long established as the primary source of early facial patterning in the vertebrate embryo [87,88,89]. These cells originate from the dorsal neural tube, delaminate, and migrate anteriorly to be incorporated into the mesenchyme of the frontonasal mass and the first pharyngeal arch. The frontonasal mass eventually gives rise to the medial and lateral nasal prominences, while the first pharyngeal arch gives rise to the maxillary and mandibular prominences [90,91] (Figure 3). 

Hh signaling is important for the survival, proliferation and differentiation of cranial neural crest cells after they have reached the craniofacial prominences [93,94]. As demonstrated by Jeong and colleagues, *Shh* is expressed in the ventral forebrain neuroepithelium, the oral ectoderm, and the pharyngeal or foregut endoderm, but is absent from the neural crest-derived mesenchyme [92,93], suggesting that neural crest cells themselves do not express *Shh*. However, the expression of *Ptch1* in both epithelium and mesenchyme suggests that Hh signaling does occur in both tissues. *Shh* signal from the foregut endoderm provides the cranial neural crest cells with information about the size, shape and orientation of the skeletal elements that will eventually form from the pharyngeal arches [95,96,97]. Blocking the *Shh* signal induces excessive apoptosis in the neural crest cells, leading to severe disruption of the facial structure [4,98]. Collectively, mutations that cause defects in neural crest cell-derived strictures are termed neurocristopathies (coined by Bolande, 1974 [99]). Neurocristopathies can arise from any genetic perturbations that result in abnormal neural crest cell development, migration or proliferation. The family of conditions involves a variety of underlying genes [100,101], among which is *Shh*. *Shh* is involved in several neurocristopathies concentrated in the craniofacial region, including holoprosencephaly, Moebius syndrome, Fetal alcohol syndrome, as well as the characteristic facial features in individuals with Down syndrome. Moebius syndrome (congenital ophthalmoplegia) patients exhibit specific cranial nerve defects including the trigeminal (V), abducens (VI) and facial (VII) nerves, with partial facial paralysis [102]. Elevated *Shh* has been implicated in the suppression of Wnt signaling in the cranial nerves, thereby affecting cranial neural crest cell survival and migration [102,103]. In individuals with Down syndrome (Trisomy 21), a characteristic craniofacial phenotype develops from an early developmental stage. Recent studies from the laboratory of Roger Reeves have linked this phenotype to reduced delamination, migration, and subsequent proliferation of neural crest cells, which appears to be due to reduced responsiveness of these cells to *Shh* [104,105]. Holoprosencephaly and fetal alcohol syndrome are discussed in Section 5 and Section 9 of this review in greater detail. 

*Shh* signaling from the craniofacial ectoderm is also involved in patterning the outgrowth and development of the facial primordia [106,107]. Expression is specifically limited to the ectoderm of the frontonasal mass and the maxillary prominences, which gives rise to the upper lip/beak and the secondary palate [107]. As mentioned above, the presence of *Ptch1* expression in both mesenchyme and epithelia of facial prominences implicates both tissues as being targets of epithelial *Shh* signaling [94]. In the ectoderm of the frontonasal mass, *Shh* and *Fgf8* expression domains define the molecular boundary of the region called the frontonasal ectoderm zone (FEZ), which is the initial site of frontonasal outgrowth [108,109]. At early stages, *Shh* expression from the forebrain acts on the cranial neural crest cells, which then induce *Shh* expression in the FEZ [110]. *Shh* from the FEZ then goes on to regulate proximodistal and dorsoventral patterning in the craniofacial complex; specifically directing outgrowth and differentiation of neural crest cell-derived skeletal structures that arise from the facial primordia [94,108,109]. Grafting experiments of the FEZ in chicken have shown that it can reprogram the developmental fate of underlying neural crest cells, inducing upper beak duplications with a dorsal-ventral polarity that reflects the orientation of the grafted tissue [108]. Thus, while expression is limited to the ectoderm, *Shh* plays a critical role in both epithelial and mesenchymal patterning of the vertebrate face.

## 5. Disorders of the Midline: Hypertelorism and Holoprosencephaly

**Figure 4:**
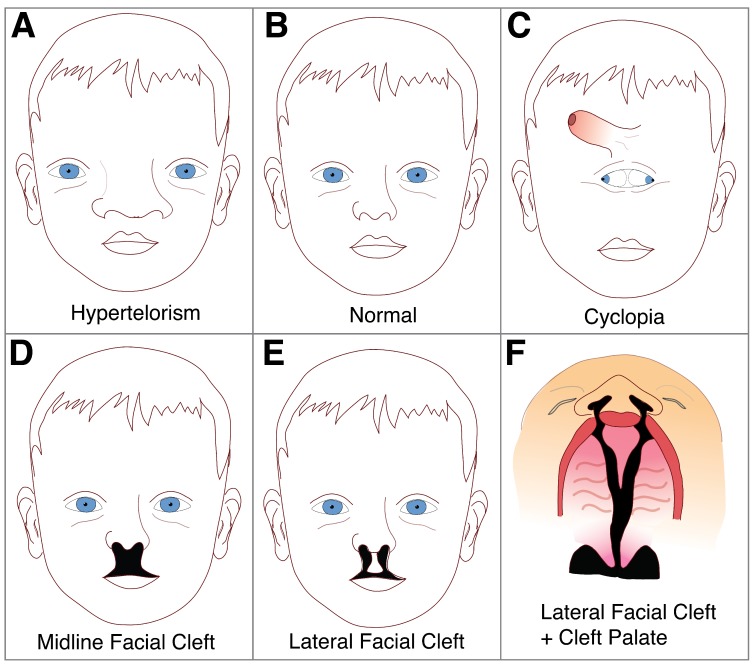
Facial appearances and associated conditions. (**A**) Hypertelorism—abnormally increased distance between eyes, often accompanied by general broadening of midline facial structures. (**B**) Normal, proportionate individual. (**C**) Cyclopia—characterized by undivided or partially divided eye fields, with displacement of the proboscis (putative nose) above the eyes. **D**. Midline Facial Cleft—facial cleft where the frontonasal mass is reduced and the maxillary prominences do not meet. (**E**) Lateral Facial Cleft—facial cleft where frontonasal mass growth is not reduced, however maxillary prominences failed to fuse. (**F**) Palatal view of a lateral facial cleft. Image adapted from Reference [117].

*Shh* is the only vertebrate Hh homolog expressed in the central nervous system [111,112], and its disruption is well documented to result in a range of craniofacial abnormalities having to do with the midline of the head [107,110,113]. Excessive Shh protein induces mediolateral expansion of the frontonasal mass, resulting in widening of the face in a condition called hypertelorism [107] (Figure 4). While wider spacing between eyes and widening of the nose generally characterize this condition, the most severe cases cause duplication of midline structures such as the nose, mouth or even the entire face [107]. Facial duplications are extremely rare but there are a few reported cases in humans and other mammals [114]. Conversely, reduced Shh protein induces collapse of the midline, leading to a condition called holoprosencephaly (HPE), which is more common and has an occurrence rate of four per 100,000 to eight per 100,000 live births [115]. In clinical studies, *Shh* mutations are found in up to 23% of affected families where HPE occurs [51,111], while approximately 25–40% exhibit cytogenetic abnormalities such as trisomy 13 or 18 [116]. While not exceedingly common, midline disorders of the face can have significant health, social and economic effects on the individual.

As early as the formation of the prechordal plate, reduction of *Shh* can lead to HPE-like narrowing of the face [118,119]. The narrowing is attributed to apoptosis of the cranial neural crest cells, which leads to a diminished frontonasal mass [118,120,121], hence its designation as a neurocristopathy. This then causes loss of ventral cell types in the forebrain, leading to incomplete cleavage of the brain and eye fields during embryogenesis [113,122]. Failure of brain cleavage can be severe (alobar, semilobar), or relatively mild (lobar), where hemispheric separation is more or less normal [123]. Alobar or semilobar HPE can lead to the formation of a single ventricle in the brain, loss of cerebral hemisphere separation, absence of corpus callosum, abnormal pituitary and thalamus, lack of olfactory lobes and optic nerves, as well as cyclopia [116,123,124]. Lobar HPE can exhibit mild craniofacial disruptions, such as ocular hypotelorism, pre-maxillary agenesis and solitary, median maxillary incisor [125]. Therefore, depending on the extent of neural crest cell loss and its effect on the forebrain, HPE can be extremely heterogeneous in its etiology [119].

Some of the more extreme (and perhaps most striking) cases of HPE involve cyclopia, which induces one, central eye field and a shift of the developing nose to a position above the eye (Figure 4C). During normal development, the optic vesicles arise from the lateral walls of the forebrain at positions that are separated by the developing structures in the ventral forebrain. In individuals with severe HPE, ventral forebrain cleavage is incomplete and optic primordia develop as a single, unpaired structure from the floor of the forebrain [126]. This then results in the characteristic single, cyclopic eye which forms centrally in the developing face, displacing the nasal structures superiorly; thus accounting for the unusual appearance of the eye developing below the nose [126] (Figure 4). In addition to cyclopia and displacement of the nasal structures, other defects may develop such as a blind-ending proboscis-like nasal structure, a single-nostril nose, as well as midline clefts in the lip, palate, nose, or all three combined [116,127]. 

Interestingly, many HPE cases do not exhibit mutations in *Shh* itself, leading to suspicion of other genes from the Hh pathway (or genes acting upon the pathway) as the likely culprits. A number of cases have now linked mutations in genes that only have a mechanistic link to the Hh pathway such as *Zic2*, *Six3*, and *Tgif* [128,129,130], as well as *Gas1* [131], *Cdo* (or *Cdon*) [132], and *Boc* [133]. Genes directly downstream of *Shh* have also been implicated in HPE, including *Ptch1* [125] and *Gli2* [134] in humans, and *Megalin* [135], *Sil* [136], and *Smo* [137] in mice. 

Another potential cause of HPE is exposure to environmental teratogens. Plant alkaloids in the jervine family have become notorious for their inhibition of the Hh pathway. They were first discovered when severe HPE (including cyclopia), was noticed in lambs in the Rocky Mountains of the western United States, where pregnant sheep grazed on the corn lily plant (*Veratrum californicum*). These plants produce two steroidal alkaloids, cyclopamine and jervine; both of which negatively affect the Hh signaling pathway [138,139]. Studies eventually found that the structural similarity of these alkaloids to cholesterol allows them to inhibit Hh signaling through binding with *Smo* directly [72,140]. By binding to *Smo*, cyclopamine and jervine negatively influence protein confirmation and cholesterol-mediated activation, ultimately inhibiting further signal transduction of the Hh pathway.

Reduced systemic cholesterol is also thought to play a role in HPE induction. For example, rodent embryos develop HPE-like features if the mothers are administered agents that induce hypocholesterolemia early in gestation [141]. Furthermore, HPE is associated with severe cases of Smith–Lemli–Opitz syndrome, where cholesterol biosynthesis is disrupted due to a mutation in an intermediary enzyme *7DHC* [142]. HPE-like features are also observed in the mouse, homozygous-null mutants of *Megalin*, as mentioned above, which is incidentally a protein involved in cholesterol transport [135]. Thus, the affiliation with cholesterols arises as a critical point at which Hh signal may be disrupted, although in many cases, the exact nature of the disruption remains to be found. These studies show that both HPE and hypertelorism arise from early disruption of Hh signaling and exhibit significant variation in penetrance, suggesting that they are dosage sensitive and may be mitigated with further research into the underlying causes. 

## 6. Cleft Lip and Palate

In amniotes (reptiles and mammals), reciprocal signaling between the mesenchyme and epithelium facilitates outgrowth and fusion of the frontonasal mass and maxillary prominences, in order to form an intact upper lip and subsequent secondary palate [92,143,144] (Figure 3). Failure of fusion results in a cleft of the lip (CL) that can potentially extend into the palate (CP). Cleft lip and/or palate (CL/P) is one of the most common congenital defects in human populations, with an incidence if 1:700–1:1000 in living newborns [1,2]. 

During the formation of the lip and secondary palate, both loss-of-function and gain-of-function of Hh signaling may result in CL/P, albeit through different mechanisms. For example, administration of the Hh antagonists vismodegib [145] and cyclopamine [146] to mouse embryos at various developmental stages induced stage-specific phenotypes that include CL/P. Both antagonists induce deficiency of the medial nasal prominences (arising from the frontonasal mass), which then fail to meet with the maxillary prominences in order to complete lip fusion. Helms et al. (1997) demonstrated a similar inhibition of Shh by teratogenic doses of retinoic acid, resulting in the inhibition of outgrowing frontonasal mass and maxillary prominence, and causing clefting of the lip and palate [147]. Similarly, the removal of Shh-expressing epithelium results in inhibition of growth in the frontonasal mass, which results in bilateral clefting [148]. In the secondary palate, epithelial to mesenchymal Shh signaling is also required for proliferative growth in the palatal shelves, disruption of which also produces cleft palate [32,94]. Thus, reduction of Shh leads to clefting in both the lip and palate, largely due to the loss of the proliferative effect that epithelial *Shh* has on the underlying mesenchyme.

Conversely, loss of *Ptch1* function in cranial neural crest cells (which then relives *Smo* inhibition and leads to constitutive activation of the Hh pathway) has been shown to cause mid-facial expansion, which culminates in cleft lip as well [149]. Additionally, Shh signaling needs to be deactivated in order to induce apoptosis in the medial epithelial edge seam cells of adjoining palatal shelves in order to facilitate fusion between them [150]. Constitutive activation of Hh signaling during palatal fusion results in persistence of the seam between adjoining palatal shelves; ultimately inhibiting complete fusion and resulting in a cleft as the embryo grows [150,151]. Similarly, mice that have ectopic *Smo* signaling in the palatal mesenchyme, and thereby gain-of-function in the Hh pathway, exhibit fully penetrant cleft palate [152]. This disruption is not only limited to the palate as Kurosaka et al. show [153]. Mice carrying a mutation of the *Ptch1* gene exhibited a persistent epithelial seam in the upper lip, in addition to hypoplastic nasal process outgrowth, which leads to a cleft in the lip. Both Kurosaka et al. and Hammond et al. propose that enhanced *Shh* signaling could result in cleft lip by negatively affecting canonical *Wnt* and *Bmp* pathways, which are also involved in fusion. Taken together, these studies show that while clefting in the lip and palate are often studied and grouped with one another, their underlying mechanisms may be radically different, even when dealing with disruption of the same genetic pathway.

## 7. Ciliopathies

As described above, it is now well established that Hh signal transduction functions through the primary cilium of the eukaryotic cell (Figure 2). The primary cilium is used to transduce molecular signals and facilitate general interaction with the environment [154,155]. Primary cilia project from the apical surfaces of cells and are composed of three main components: an axoneme, a basal body and a ciliary membrane. The axoneme consists of a ring of nine microtubule doublets that form the core of the cilium, the basal body is a microtubule-based structure that anchors the cilium to the cell body, and the ciliary membrane is a specialized membrane that covers the cilium [156] (Figure 2). Externally, the ciliary membrane harbors receptors for a number of signaling pathways, making the primary cilium an important regulator of developmental signaling from the Hh and Wnt pathways, to Platelet-derived growth factor (PDGF) alpha and Polycystin [157]. Studies have found that mice with phenotypic characteristics of Hh signaling defects often harbor mutations in ciliogenesis genes rather than the Hh pathway itself [158,159]. Therefore, craniofacial defects that are attributed to abnormal Hh signaling may be secondary to ciliary dysfunction. 

When cilial development or function is disrupted, the resulting disorders are classified as ciliopathies [160,161]. Over 100 conditions in humans are either known or suspected to fall under this category [162]. Because the majority of cells in the body have cilia, ciliopathies exhibit an extensive range of clinical manifestations [160,163,164]. Craniofacial ciliopathies range in phenotype from subtle midline defects to fully penetrant orofacial clefts [164]. Along with individual conditions, the aforementioned craniofacial pathologies are also involved in a number of condition with syndromic backgrounds, including but not limited to: Oro-facial-digital syndrome (OFDS; OMIM 311200), Joubert syndrome (JBTS; OMIM 213300), Bardet-Biedl syndrome (BBS; OMIM 209900), Jeune asphyxiating thoracic dystrophy (JATD; OMIM 208500), Short rib-polydactyly syndromes (SRPS; OMIM 613091, 263520), Meckel-Gruber Syndrome (MKS; OMIM 249000), Ellis–van Creveld syndrome (EVC; OMIM 225500), and Cranioectodermal dysplasia (CED; OMIM 218330) [154,164,165]. It is relevant to note that these syndromes exhibit a variety of phenotypes; many of them overlapping with those seen during Hh signal disruption.

Ciliopathies arise through mutations in a number of different genes that ultimately share the distinction of causing failure of cilial development or function. The effects of ciliopathies on Hh signaling are often contradictory and convoluted. For example, in some studies, mutations seem to induce loss of Hh signaling, while in others, Hh signaling appears to increase. This is particularly evident in craniofacial phenotypes, where loss of cilia can lead to narrowing of the head (associated with loss of Hh) as well as incidences of widening of the mid-face (associated with gain of Hh) [165,166]. For example, animals with a defective basal body protein RPGRIP1L produce fewer cilia, which in turn causes inefficient transduction of *Shh* signaling, causing defects consistent with loss of Hh signaling (Zaghloul et al., 2011, references within [77]). Conversely, loss of cilia in murine neural crest cells through inactivation of *Kif3a* enhances Hh signaling in the face, causing increased neural crest cell proliferation in the facial prominences and inducing widening of the face [165]. These differences may be due to the specific component of the Hh pathway that is disrupted, such as mutation in the anterograde vs. retrograde transport systems. 

Intraflagellar transport proteins (IFTs) are also involved in ciliopathic disruption of Hh signaling. IFTs are a highly conserved family of multimeric proteins found in ciliated cells and are essential for ciliogenesis as well as shuttling of proteins to and from the cytoplasm [167,168]. Inside the cilium, they shuttle downstream Hh pathway components such as *Smo* and *Gli* through the cilium in a highly regulated manner during signal transduction [158]. Using mutant mouse lines, ift172, ift88, kif3a, and dync2h1 were identified as critical components of *Shh* signaling through the primary cilium [158,169]. Subsequently, many more IFT mutations have been characterized and shown to induce phenotypes similar to *Shh* signal disruption, likely due to loss of anterograde-retrograde transport of Hh signaling components [170,171,172,173,174]. Interestingly, there seem to be differences between anterograde and retrograde IFT protein mutations and their effect on Hh signaling. Ift88^−/−^ or Ift172^−/−^ embryos lack anterograde transport and exhibit reduced *Shh* signaling [158]. In contrast, Ift139^−/−^ and Ift122^−/−^ lack retrograde transport and display excessive *Shh* signaling [174,175]. Once again, differences in the phenotype may have to do with the specific components of the Hh pathway that these IFTs are transporting, warranting further study of these systems in the future.

## 8. Talpid Chicken Mutants 

The talpid chicken mutants are some of the more famous cases of Hh-related ciliopathy in craniofacial development. *Talpid*, *talpid^2^*, and *talpid^3^* are distinct, embryonic lethal, autosomal recessive mutant chicken lines that arose independently and exhibit malformation in the face and limb [176]. They received the name “talpid” due to their polydactylous forelimb, reminiscent of the members of the Talpidae family of mammals (e.g., moles and shrews). Randall Cole identified the original *talpid* mutant in 1942. *Talpid* had a severely shortened upper beak and shortened mandible, while posterior structures such as the eyes seemed normal [177]. Unfortunately, the original *talpid* line is now extinct [176]. 

Ursula Abbott, Lewis Taylor, and Hans Abplanalp subsequently described *talpid^2^* in 1960 at the University of California, Davis. *Talpid^2^* exhibits a less severe mutation than *Talpid*, with a short and broad frontonasal mass and underdeveloped maxillary prominences that resulted in a shortened upper beak with bilateral clefting, hypoglossia, and outgrowths on the jaw that resembled tooth-like structures [165,178]. Genetic analysis has identified a 19 base pair deletion in the C2 calcium-dependent domain containing 3 (*C2cd3*) gene [178]. *C2cd3* is essential for ciliogenesis and localizes near the distal tip of centrioles (such as the basal body) and physically interacts with other centriolar and IFT proteins [179,180,181]. *C2cd3* also underlies human oral-facial-digital syndrome [176,180], and is an essential regulator of intracellular transduction of Hh signaling. 

Various studies have reported aberrant *Shh* expression in the *talpid^2^* mutant [182,183,184]. The expanded midline and cleft upper beak are traditionally associated with gain of *Shh* function in the face [107]. More recently, Chang and colleagues identified increased *Shh* and *Gli3A* in the frontonasal mass, implicating *Gli3A* activity as the fundamental cause of the *talpid^2^* facial phenotypes [178]. Interestingly, they also found that while *Shh* expression was increased in both the frontonasal mass and maxillary prominence, *Ptch1* expression was reduced in the frontonasal mass, while remaining the same in the maxillary prominence. This suggests these mutants harbor an unexplained decoupling between the Hh ligand and its traditional receptor, *Ptch1*.

*Talpid^3^* embryos, on the other hand, exhibit an HPE-like reduced midline, with the reduction and displacement of the frontonasal mass [185,186]. Later analysis identified a single thymine insertion in the previously uncharacterized *Kiaa0586* gene, resulting in a frame-shift and premature stop codon [187]. The KIAA0586 protein, eventually named TALPID3, was isolated in a proteomic analysis [188], and was localized to a ring at the distal end of the basal body [189]. Loss of TALPID3 resulted in a loss of non-motile and motile cilia in both chicken and zebrafish [190,191,192,193]. Mutations in the human ortholog of *Talpid^3^* have recently been associated with Joubert Syndrome, a ciliopathy with a very similar craniofacial phenotype to both *talpid^3^* and Hh signal disruption [194,195,196]. 

Indeed, *Talpid^3^* mutants exhibit a complete loss of *Shh* and *Ptch1* expression in the ventral forebrain and developing craniofacial complex [187,197]. The midline collapses due to an underdevelopment of the frontonasal mass, resulting in the direct fusion of the two maxillary prominences with each other [176]. Interestingly, Davey and colleagues report that in wing buds of *talpid^3^* animals, levels of *Gli3A* are markedly increased similar to *talpid^2^*, despite loss of *Shh* expression [187]. Given that both mutants exhibit similar ciliary phenotypes however, it is unclear what is the basis for difference in *Shh* expression. One must also consider the fact that other pathways (e.g., *Wnt*) are mediated though primary cilia and may play a role in the aberrant behavior of Hh signaling as well as the phenotypes exhibited by the *talpid^2^* and *talpid^3^* mutant lines. Recently, Matsubara et al. have identified a new talpid mutant type in the Japanese quail, which may help to shed light on the various peculiarities of the talpid lines [198].

## 9. Fetal Alcohol Syndrome 

Hh signaling is also involved in the developmental phenotype associated with fetal alcohol syndrome (FAS). FAS is a congenital disorder in the developing embryo that is attributed to the consumption of alcohol by the pregnant mother. It is characterized by an array of developmental abnormalities that include, among others, craniofacial malformations [199]. Estimates of global FAS occurrence ranges from 2 in 100,000 to as high as 5.4 in 1000 people [200]. The phenotypic manifestations of FAS are variable, with many individuals exhibiting abnormal brain morphogenesis and accompanying craniofacial defects [201,202,203]. Deficiencies in developmental processes associated with FAS include increased apoptosis [204], cell adhesion defects [205], accumulation of free radicals [206], disruption of growth factors [207], and altered retinoic acid biosynthesis [208]. In severe cases, the abnormalities of the face and brain fall within the spectrum of HPE [209]. Fortunately, model organisms such as chickens also exhibit similar craniofacial phenotypes upon alcohol exposure, including HPE, hypotelorism, and micrognathia, allowing for access to the condition in a laboratory setting [210,211,212]. 

Developmental defects associated with FAS are attributed to increased apoptosis of cranial neural crest cells, a known consequence of Hh signal disruption [93,213,214]. The neural crest cells that do not undergo apoptosis, migrate significantly shorter distances and exhibit a disorganized cytoskeleton with fewer filopodia, lamellipodia, and focal adhesions [215,216]. Studies in a number of species have also shown that the neural crest cells death observed after ethanol exposure is comparable to blocking *Shh* signaling [93]. Both processes result in a reduction in head size and apoptosis of cranial neural crest cells, which results in reduced frontonasal mass size and pharyngeal arches [93]. Importantly, Ahlgren and colleagues have shown that exogenous *Shh* enhances cranial neural crest cell survival within the facial primordial, thereby mitigating the FAS phenotype [93]. These experiments help to functionally link FAS with Hh signaling, as well as identifying a putative point of intervention for the disease. 

A more upstream mode of interaction between the Hh pathway and FAS seems to involve the dependence of Hh ligands on cholesterol modification. In a study conducted by Li et al., alcohol was shown to interfere with the modification of *Shh* by cholesterol, thereby inhibiting association of *Shh* with the lipid rafts in the cell membrane that facilitate extracellular transport of the signal molecule [213]. Furthermore, alcohol exposure results in a dose-dependent decrease of total cholesterol content in zebrafish embryos, consequently inducing a dose-dependent decrease in cholesterol-modified Shh protein [213]. This then explains the association between alcohol concentration and the broad spectrum of developmental defects that characterize FAS. Moreover, when alcohol-exposed embryos are supplemented with cholesterol, the phenotype is mitigated, further implicating reduction of cholesterol as a major player in FAS phenotype generation [213]. Of course, ligand modification is not the only point at which cholesterol plays a key role in Hh signaling. Cholesterol has also been shown to bind *Smo* directly and activate it [73,74]. Thus, the FAS phenotype could also arise due to the loss of *Smo* activity from the reduction of cholesterol. Li et al predicted this scenario, whereby exogenous cholesterol may also directly stimulate *Smo* and initiate Hh signal transduction, independent of the Hh ligand [213]. Thus, a potential next step in assessing this interaction is characterization of cholesterol-modified Shh and cholesterol-modified Smo concentrations in FAS animals supplemented with cholesterol, in order to discern which protein the extra cholesterol is associating with. 

Yet another interaction between FAS and Hh signaling may be a direct effect of alcohol on gene expression. A number of studies have demonstrated downregulation of the Hh pathway both through genes downstream of the Hh ligand (e.g., *Ptch1*, *Gli2*, *Gli3*) as well as *Shh* itself [93,217,218]. This may in turn inhibit directed migration of cranial neural crest cells toward the *Shh* source in the pharyngeal arch epithelium, since expression is significantly reduced [93,218]. Otherwise, direct depression of *Shh* expression could also be the reason for apoptosis in neural crest cells, reducing overall size of the face.

Finally, the background genotype of the individual may also play a role in the predisposition and severity of FAS phenotype. Studies of monozygotic twins have demonstrated 100% concordance for FAS between siblings, while only 7/11 dizygotic twins displayed concordance [219,220]. This provided precedent for work on identifying the differential susceptibility of certain genotypes to FAS. For example, while *Shh^−/−^* mice exhibit severe HPE [113] and *Gli2^−/−^* mice develop a number of craniofacial defects [221,222], while *Shh^+/−^* and *Gli2^+/−^* mice develop relatively normal [134,222]. However, ethanol exposure resulted in 3.2 and 6.6 fold increase respectively in the severity of phenotype for *Shh^+/−^* and *Gli2^+/−^* fetuses, when compared to wild type littermates [209]. A similar scenario occurs with the *Cdon* gene, which functions as a co-receptor for the *Shh*, assisting in activation of the Hh pathway. 129S6.*Cdon^−/−^* mice generally exhibit a low penetrance of microform HPE [223]. However, the genotype is highly sensitive to in utero ethanol exposure. Ethanol-exposed *Cdon* mutant mouse embryos exhibit defects in *Shh* signaling, as well as craniofacial defects commonly attributed to Hh signal disruption like palatal clefting, and an increase in lobar HPE by 50%. This, while wild-type and heterozygote littermates largely exhibited normal phenotype [223]. In a later study, the same group found that 129S6.*Cdon ^−/−^* mice failed to reach a threshold level of *Shh* signaling and decided to create mice that were deficient in *Ptch1* (to increased Hh signaling), in order to mitigate the lack of Shh signal. *Cdon ^−/−^;Ptch1^+/−^* embryos exhibited significantly reduced penetrance of HPE [224]. These finding lend strong support to the notion that mutations associated with the Hh pathway that may otherwise lead to a relatively normal phenotype, predispose the individual for an amplified effect from prenatal ethanol exposure. 

## 10. Statins and Cholesterol Biosynthesis

In general, organisms depleted of lipids and sterols by treatment with the drugs that sequester hydrophobic molecules such as cyclodextrin, or with statins, exhibit facial defects consistent with Hh pathway disruption [225]. Statins are a group of drugs used to treat hypercholesterolemia by pharmacologically reducing cholesterol biosynthesis [226]. When taken during the first trimester of pregnancy, statins have been shown to interfere with normal development of limb and central nervous system, inducing conditions such as HPE [227]. Moreover, genetic conditions where cholesterol biosynthesis is disrupted (e.g., Smith–Lemli–Opitz syndrome, desmosterolosis) or bioavailability is otherwise compromised, also share many symptoms with Hh signal disruption [228]. Initial hypotheses about the nature of this association focused on the reliance of Hh protein on posttranslational modification with cholesterol, and its disruption. However, this does not seem to be the case, since exogenous Shh does not ameliorate the developmental defects associated with exposure to statins or cyclodextrin [71,225].

Instead, it appears that interruption in Hh pathway transduction comes from a decreased response to the Hh ligand, as opposed to an inability for the ligand to signal to its receptors [138]. Furthermore, Hh protein processing proceeds normally in cells with genetic deficiencies in sterol synthesis [225]. In fact, work by Cooper et al. (2003) shows that sterol depletion affects *Smo* directly, as opposed to the *Shh* ligand, which was efficiently processed despite cyclodextrin treatment or growth in lipid-depleted culture medium. Sterols are able to deplete wild-type function, while an oncogenic, activated *Smo* mutant appeared to be resistant [225]. This suggested that *Smo* conformation may be the target of cholesterol deprivation. This is not surprising considering the fact that studies have recently recognized that *Smo* is indeed bound and activated by cholesterol during Hh signal transduction [73,74]. Interestingly, Roux and colleagues showed that a hypercholesterolemia-provoking diet can be effective for preventing HPE induced by inhibitors of cholesterol synthesis, although it was found to increase overall fetal mortality and the formation of other malformations [229]. Maternal hypercholesterolemia (either permanent or temporary), was also found to induce formation of fatty streaks in the fetal aorta of younger fetuses [230], which my proceed into childhood and adulthood [231,232]. Therefore, unlike in the case of FAS, developmental defects induced by cholesterol depletion have been pinpointed to one cause: lack of *Smo* activation. This then allows for a reasonable prospect at treating the condition in the developing embryos. That said, treatment may not be as simple as increasing cholesterol since this is shown to cause its own health problems.

## 11. Craniosynostosis

Besides the developing face, cranial bones are also affected by disruption in Hh signaling. The calvarium (cranial vault) forms from a combination of paraxial mesoderm and newly migrated cranial neural crest cells [233,234]. In tetrapods, the calvarium is mainly composed of paired frontal and parietal bones, with lesser contribution from postparietal bones [233,235]. These bones arise through intramembranous ossification at multiple centers and expand to meet each other at fibrous joints (sutures) that remain patent through adolescence [236]. Sutures contain proliferative osteogenic precursors that differentiation into osteoblasts, forming new bone as the brain and skull grow [117,235,236]. Upon completion of growth, a signal from the underlying dura mater induces cells at the growing edge to differentiate into osteoblasts, eventually connecting the two adjacent bone plates end-to-end [235,236].

Suture cells have to differentiate in order to grow the bony plates, while at the same time remain undifferentiated in the center of the suture to allow for suture patency, making for a delicate balance between differentiation and proliferation [236]. In some cases, the balance is disrupted and premature fusion can take place within the cranial sutures, resulting in a condition called craniosynostosis (coined by Adolph Otto in 1830 [236]). Synostosis prevents expansion of the growing calvarium components and accommodation of neurocranial growth, and can lead to craniofacial dysmorphology and impairment in the central nervous system such as: elevated intracranial pressure [237], learning disabilities [238,239], and impaired eyesight [240]. Due to the severe of the aforementioned symptoms, craniosynostosis receives significant interest from the academic and biomedical community.

Hh signaling has recently become a focus of investigation in studies of cranial suture biology since both *Shh* and *Ihh* are known regulators of osteogenesis [241]. Studies of synostotic models where *Ihh* protein levels are increased indicate a positive correlation between *Ihh* overexpression and craniosynostosis [242,243]. *Ihh* is expressed in mature calvarial osteoblasts [244,245], and is thought to function as a pro-osteogenic factor that induces intramembranous ossification though induction of *Ptch* and *Bmp2/4* expression [246,247], *Ihh^−/−^* mice on the other hand, exhibit reduction of osteogenic markers, reduced *Bmp2/4* expression, as well as generally reduced expansion and thickness of calvarial bones with widened cranial sutures [247,248]. More recent work by Vesitinen and colleagues established *Ihh* as the functional ligand for Hh regulated osteogenesis in the calvaria [245]. On the other hand, the exact role of *Shh* in cranial osteogenesis seems to elude clarification thus far, with the precise expression pattern of *Shh* in the cranial suture not yet agreed upon [241,247]. While *Shh* is expressed in developing cranial bones [249], its functional role in this tissue has not been evaluated since *Shh^−/−^* embryos die prior to cranial osteogenesis [113]. However, the predominant presence of *Shh* in the suture mesenchyme and its overlapping expressing pattern with mesenchymal cell proliferation has led authors to postulate that *Shh* has a role in maintaining suture patency [247,249], although this has yet to be shown experimentally. 

## 12. Conclusions

The versatility of the Hh pathway has allowed it to occupy many niches within the patterning of the vertebrate embryo. Alas, this has also magnified the number and variety of developmental disorders it is associated with. Fortunately, work in the last few decades has delineated the fundamentals of Hh signaling. The field has made great advancement in formulating a better understanding of the interaction between the Hh ligand, *Ptch1*, and *Smo*, which were rather nebulous until the last decade or so. Studies with constitutively activated *Smo* have allowed for a better understating of the relationship it has with *Ptch1* as well as with cholesterol molecules. More recently, application of digital technologies such as computer-aided modeling have also delineated the physical structure of PTCH1 as a protein pump (summarized by Sommer and Lemmon (2018) (Cite 77)), confirming some of the molecular and biochemical hypotheses regarding its binding to the Hh ligand as well as its inhibition of *Smo*. 

With the fundamental mechanisms of the pathway increasingly understood, focus may be turned towards specific cases of disruption, like those associated with craniofacial pathologies. For example, some conditions such as craniosynostosis have received very little attention in terms of our understanding of the role of Hh signaling, although studies of expression patterning have provided tantalizing clues. Yet other conditions, such as HPE or ciliopathies have been studied and described in greater detail with respect to their fundamental causes, yet variation in penetrance of these conditions remains poorly understood. Fortunately, we have established laboratory protocols to induce the aforementioned conditions, whether through exposure to teratogens in the case of HPE or KO mice in the case of ciliopathies. We can now use these to tease apart some of the variability. For instance, there is strong evidence linking Hh co-receptors to HPE (e.g., Cdon, Gas1, Boc) in the craniofacial region and elsewhere, which seem to play substantial roles in susceptibility to Hh signal disruption as well as penetrance. Further study of these genes thorough large-scale genomic studies could fine-tune our understanding of the phenotypes that we observe across affected individual. Similar focus on the various IFT proteins is also likely to yield a better understanding of the variability that is inherent in ciliopathies.

Lastly, as we achieve a better understanding the mechanisms behind the effects of teratogens, it is becoming obvious that there is a common thread across the various molecules: they are most often affecting the physiological coupling of the Hh pathway with cholesterol. Teratogens such as alcohol or cyclopamine directly exploit this aspect of Hh signal transduction, whether in the ligand itself or in *Smo*. Observing these overarching patterns will be important in moving forward towards mitigating the severity of these conditions associated with Hh signaling and identifying other possible points of interference and treatment.
